# Supplementary material for: Copolymers Derived from Two Active Esters: Synthesis, Characterization, Thermal Properties, and Reactivity in Post-Modification
Source: Molecules. 2022 Oct 12;27(20):6827. doi: 10.3390/molecules27206827 (PMC9607591; doi:10.3390/molecules27206827)
Supplement: Supplementary file 1 [file molecules-27-06827-s001.zip › molecules-1941443-supplementary.pdf]

Supplemental Information

Copolymers derived from two active esters: Synthesis, Characterization, Thermal Properties and Reactivity in Post-modification

Thi Phuong Thu Nguyen, [a] *Nadine Barroca-Aubry,* [a] *Caroline Aymes-Chodur,* [a] *Diana Dragoe,* [a] *Gaëlle Pembouong,* [b] *Philippe Roger* [a]*

[a] Institut de Chimie Moléculaire et des Matériaux d'Orsay (ICMMO), UMR 8182, Université Paris-Saclay, CNRS, 91405, Orsay, France

[b] Equipe Chimie des Polymères, Institut Parisien de Chimie Moléculaire, Sorbonne Université, CNRS, 4 Place Jussieu, 75005 Paris, France

Corresponding Author

* Corresponding author: philippe.roger@universite-paris-saclay.fr

--

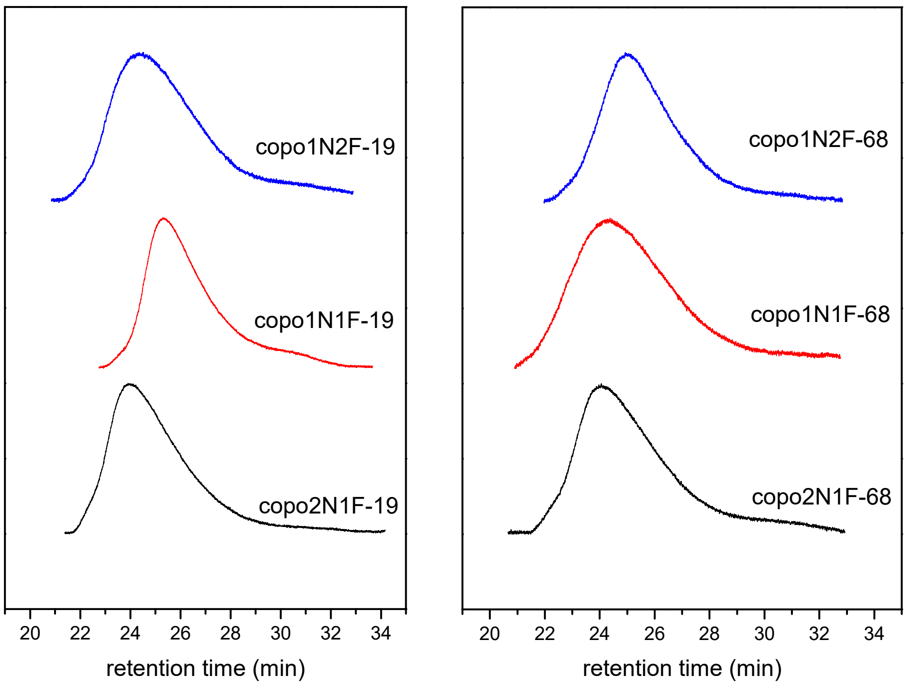

Figure S1. RI detector responses obtained by SEC in DMF/LiBr after 19 and 68 hours of polymerization for various copolymers

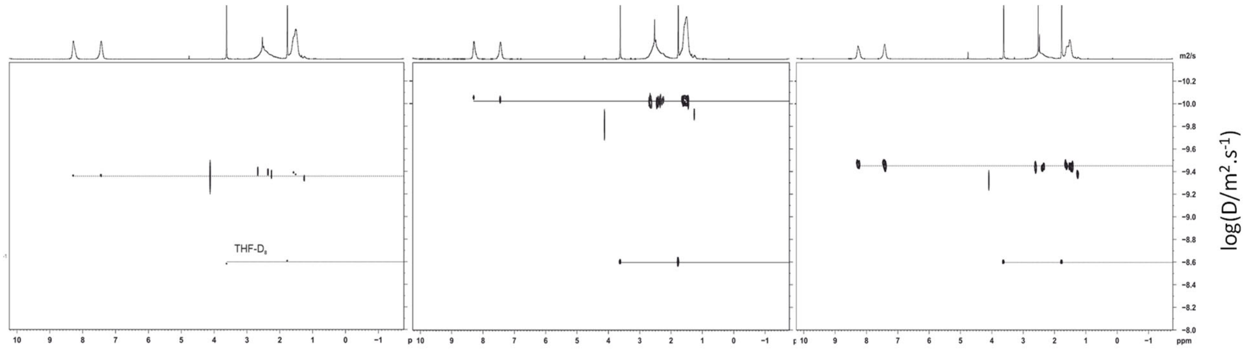

TNB90-1N1F
D= 4.27 x 10^{-10} $m^2.s^{-1}$

TNB90-1N2F
D= 9.64 x 10^{-11} $m^2.s^{-1}$

TNB90-2N1F
D= 3.52 x 10^{-10} $m^2.s^{-1}$

Figure S2. DOSY spectra of copolymers obtained after 68 hours of polymerization.

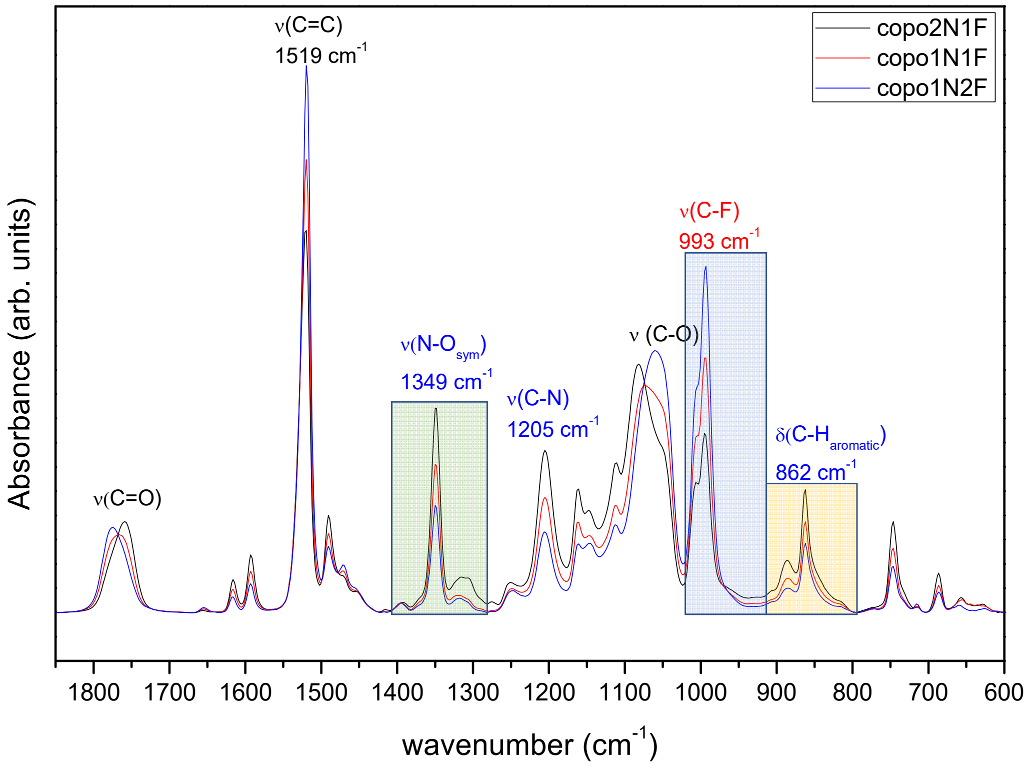

Figure S3. Superimposed ATR-FTIR spectra of three copolymers obtained at 68 hours polymerization. The highlighted boxes indicate the area from which integration was taken for the elaboration of Figure 2d and Figure 2e.

Table S1. Summary of absorption peaks of PPFPMA, PNPMA and their copolymers

**Entry**	Assignment	**λ (cm^{-1}) PPFPMA**	**Copo1N2F** f_{PFPMA} = 0.67	**Copo1N1F** f_{PFPMA} = 0.50	**Copo2N1F** f_{PFPMA} = 0.33	**λ (cm^{-1}) PNPMA**
**1**	$\upsilon_{C=O}$	1778	1772 cm^{-1}	1766 cm^{-1}	1757 cm^{-1}	1752
**2**	$\upsilon_{C=C}$	1517	1519 cm^{-1}	1519 cm^{-1}	1519 cm^{-1}	1523
**3**	υ_{N-O}	-	1350 cm^{-1}	1350 cm^{-1}	1350 cm^{-1}	1350
**4**	υ_{C-N}	-	1205 cm^{-1}	1205 cm^{-1}	1205 cm^{-1}	1205
**5**	υ_{C-O}	1056	1058 cm^{-1}	1076 cm^{-1}	1083 cm^{-1}	1089
**6**	υ_{C-F}	993	993 cm^{-1}	993 cm^{-1}	993 cm^{-1}	-
**7**	δ_{C-H}	-	862 cm^{-1}	862 cm^{-1}	862 cm^{-1}	862

Table S2. Atomic number percentages and atomic mass percentages of copolymers derived from PFPMA and NPMA calculated from NMR, XPS and elemental analysis results.

	Atomic Number Percentage (%)						Atomic Weight Percentage (%)								
	NMR			XPS			XPS			NMR			Elemental Analysis		
	2N1F	1N1F	1N2F	2N1F	1N1F	1N2F	2N1F	1N1F	1N2F	2N1F	1N1F	1N2F	2N1F	1N1F	1N2F
%C	64.0	62.0	61.0	65.0	62.0	62.0	57.8	53.1	52.7	52.9	51.5	50.1	54.0	52.3	50.9
%N	4.0	3.0	2.0	4.0	3.0	2.0	4.2	3.0	2.0	4.0	2.9	1.9	4.3	3.0	2.0
%O	22.0	18.0	16.0	20.0	17.0	15.0	22.5	19.4	17.0	23.3	20.5	17.9	-	-	-
%F	10.0	17.0	21.0	11.0	18.0	21.0	15.5	24.4	28.3	14.4	20.5	26.3	-	-	-
C/N	16.0	20.7	30.5	16.3	20.7	31.0	13.9	17.7	26.6	13.3	17.8	25.9	12.7	17.3	25.8
C/O	2.9	3.4	3.8	3.4	3.7	4.1	2.6	2.7	3.1	2.3	2.5	2.8	-	-	-
N/F	0.4	0.2	0.1	0.4	0.2	0.1	0.3	0.1	0.1	0.3	0.1	0.07	-	-	-

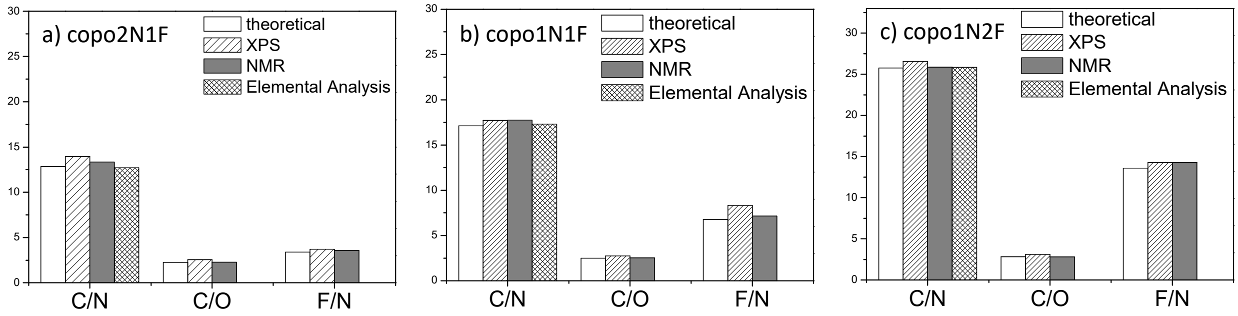

Figure S4. Comparison of atomic weight ratios obtained by different analyses

Table S3. Details on fitting parameters for deconvolution of different atomic core levels.

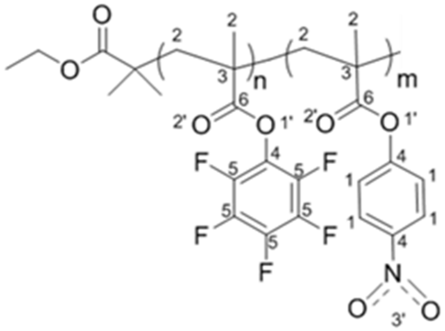

Core level	Bond assignment		Copolymers								
			Copo1N2F			Copo1N1F			Copo2N1F		
			BE	FWHM	%	BE	FWHM	%	BE	FWHM	%
C1s	C=C	1	284.5	1.60	13.3	284.6	1.70	21.1	284.6	1.54	22.6
	C-C	2	285.0	1.34	20.0	285.0	1.27	21.1	285.0	1.29	24.5
	C*-CH_3	3	285.8	1.20	13.3	285.6	1.01	10.5	285.7	1.07	11.3
	C-O, C-N	4	286.8	1.40	10.0	286.3	1.22	10.5	286.4	1.21	11.3
	C-F	5	287.9	1.69	33.4	287.7	1.65	26.3	287.6	1.67	18.9
	COO	6	289.2	1.65	10.0	288.9	1.56	10.5	288.8	1.58	11.3
O1s	NO_2	1'	532.3	1.72	25.4	532.4	1.75	32.3	532.3	1.87	38.24
	C=O	2'	533.2	2.50	40.7	532.9	2.47	35.5	533.1	2.00	32.35
	C-O	3'	534.2	2.52	33.9	534.1	2.18	32.2	534.3	2.25	29.41
N1s	NO_2		405.9	2.14	100	405.8	1.90	100	405.8	1.92	100
F1s	C-F		688.2	2.02	96.7	688.1	1.85	96.1	688.1	1.88	96.9
	Shake up		695.3	2.02	3.3	695.0	2.27	3.9	695.1	1.91	3.1

Table S4. Tg of copolymers calculated by Gordon-Taylor equation based on Tg of homopolymers

$$T_{g,co} = \frac{\omega_1 \times T_{g,1} + K \times \omega_2 \times T_{g,2}}{\omega_1 + K \times T_{g,2}}$$

**Entry**		$\mathbf{F_{PFPMA}}$	$\mathbf{F_{NPMA}}$	**weight fraction**		**Tg**	
				**PFPMA**	**NPMA**	Tg (K)	Tg (°C)
1	PPFPMA	1	0	1.000	0.000	390.75	**117.6**[a]
2	Copo1N2F-68	0.70	0.30	0.739	0.261	399.12	**141.7**[a] (126.5[b])
3	Copo1N1F-68	0.50	0.50	0.549	0.451	405.47	**147.9**[a] (133.0[b])
4	Copo2N1F-68	0.32	0.68	0.364	0.636	411.83	**154.2**[a] (139.4[b])
5	PNPMA	0	1	0.000	1.000	424.95	**151.8**[a]

[a] values obtained from experimental DSC measurements

[b] values calculated from Gordon-Taylor equation based on experimental Tg of homopolymers

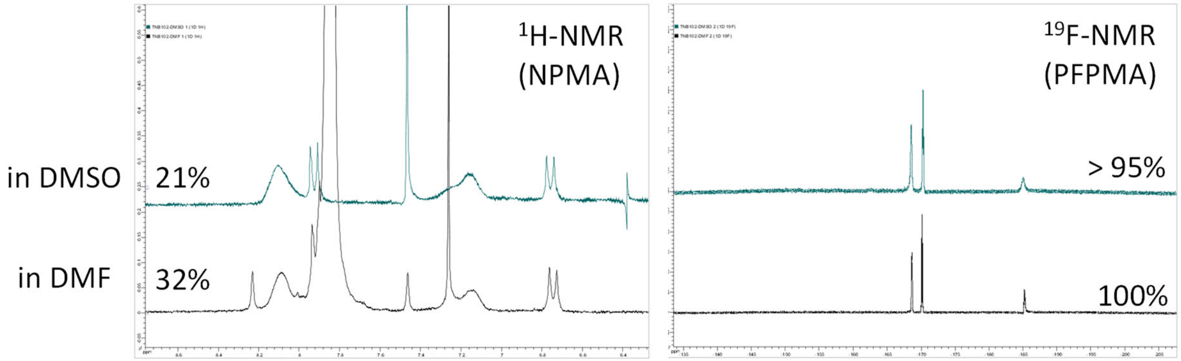

Figure S5. NMR spectra (^{1}H: left, ^{19}F: right) of post-modification of copo1N1F with citronellol in DMSO (top) and DMF (bottom)
